# Nurses' Work for the Poor

**Published:** 1920-12-18

**Authors:** 


### Nurses' Work for the Poor.

RECENTLY, at the Howard de Walden Club, 35 Langham Street, W., a very cheerful company of nurses met for tea and to inspect the work of the Nurses' Needlework Guild. Some four hundred garments had been sent in, and did the workers credit, for they were well cut, well sewn, and particularly warm. The babies' clothes, as usual, were the most fascinating. The work goes to the poorest hospitals and districts in London, and will be much appreciated this Christmas, when clothes are almost prohibitive in price. When one remembers the long hours of nurses on duty, their slender means, and family claims, it is a matter for wonder that they should find time and money for this work of true charity. Yet year after year they do, and perhaps only the profession know how greatly it is appreciated by the patients to whom they have already rendered other service by day and night.

WE regret that in reviewing the new handbook on "The Theory and Practice of Nursing," written by Miss Gullan, sister-tutor at St. Thomas's, and published by Messrs. H. K. Lewis & Co., we quoted the price as 12s. 6d. net, whereas it is, in fact, 10s. 6d.

